# Supplementary material for: Comparative Mitogenomics and Phylogenetic Implications for Nine Species of the Subfamily Meconematinae (Orthoptera: Tettigoniidae)
Source: Insects. 2024 Jun 3;15(6):413. doi: 10.3390/insects15060413 (PMC11204050; doi:10.3390/insects15060413)
Supplement: Supplementary file 1 [file insects-15-00413-s001.zip › Schedule/Figure S6. Phylogenetic tree obtained from BI analysis based on 13 PCGs + 2rRNA.pdf]

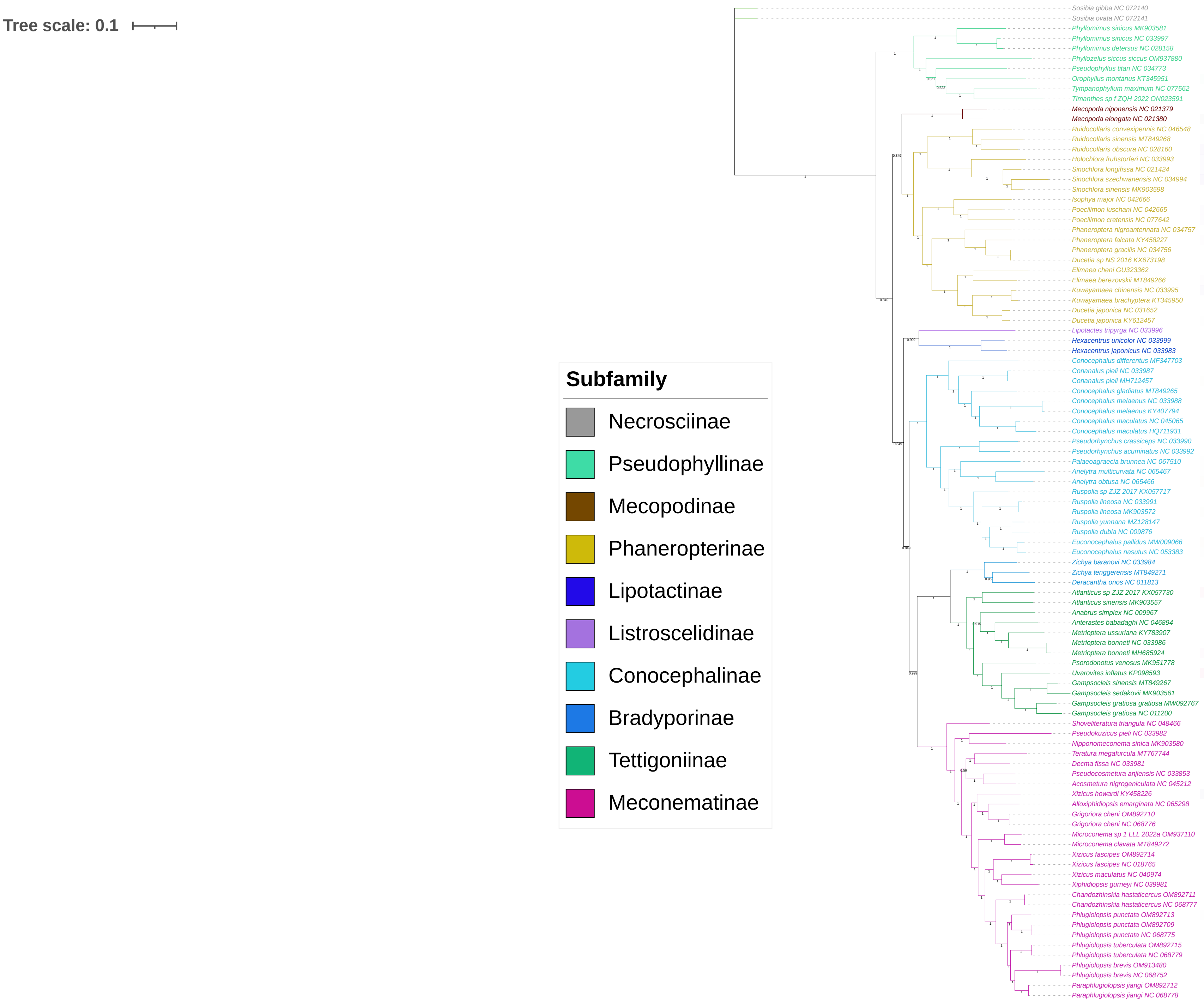
Tree scale: 0.1
Subfamily
Necrosciinae
Pseudophyllinae
Mecopodinae
Phaneropterinae
Lipotactinae
Listroscelidinae
Conocephalinae
Bradyporinae
Tettigoniinae
Meconematinae
Sosibia gibba NC 072140
Sosibia ovata NC 072141
Phyllomimus sinicus MK903581
Phyllomimus sinicus NC 033997
Phyllomimus detersus NC 028158
Phyllozelus siccus siccus OM937880
Pseudophyllus titan NC 034773
Orophyllus montanus KT345951
Tympanophyllum maximum NC 077562
Timanthes sp f ZQH 2022 ON023591
Mecopoda niponensis NC 021379
Mecopoda elongata NC 021380
Ruidocollaris convexipennis NC 046548
Ruidocollaris sinensis MT849268
Ruidocollaris obscura NC 028160
Holochlora fruhstorferi NC 033993
Sinochlora longifissa NC 021424
Sinochlora szechwanensis NC 034994
Sinochlora sinensis MK903598
Isophya major NC 042666
Poecilimon luschani NC 042665
Poecilimon cretensis NC 077642
Phaneroptera nigroantennata NC 034757
Phaneroptera falcata KY458227
Phaneroptera gracilis NC 034756
Ducetia sp NS 2016 KX673198
Elimaea cheni GU323362
Elimaea berezovskii MT849266
Kuwayamaea chinensis NC 033995
Kuwayamaea brachyptera KT345950
Ducetia japonica NC 031652
Ducetia japonica KY612457
Lipotactes tripyrga NC 033996
Hexacentrus unicolor NC 033999
Hexacentrus japonicus NC 033983
Conocephalus differentus MF347703
Conanalus pieli NC 033987
Conanalus pieli MH712457
Conocephalus gladiatus MT849265
Conocephalus melaenus NC 033988
Conocephalus melaenus KY407794
Conocephalus maculatus NC 045065
Conocephalus maculatus HQ711931
Pseudorhynchus crassiceps NC 033990
Pseudorhynchus acuminatus NC 033992
Palaeoagraecia brunnea NC 067510
Anelytra multicurvata NC 065467
Anelytra obtusa NC 065466
Ruspolia sp ZJZ 2017 KX057717
Ruspolia lineosa NC 033991
Ruspolia lineosa MK903572
Ruspolia yunnana MZ128147
Ruspolia dubia NC 009876
Euconocephalus pallidus MW009066
Euconocephalus nasutus NC 053383
Zichya baranovi NC 033984
Zichya tenggerensis MT849271
Deracantha onos NC 011813
Atlanticus sp ZJZ 2017 KX057730
Atlanticus sinensis MK903557
Anabrus simplex NC 009967
Anterastes babadaghi NC 046894
Metrioptera ussuriana KY783907
Metrioptera bonneti NC 033986
Metrioptera bonneti MH685924
Psorodonotus venosus MK951778
Uvarovites inflatus KP098593
Gampsocleis sinensis MT849267
Gampsocleis sedakovii MK903561
Gampsocleis gratiosa gratiosa MW092767
Gampsocleis gratiosa NC 011200
Shoveliteratura triangula NC 048466
Pseudokuzicus pieli NC 033982
Nipponomeconema sinica MK903580
Teratura megafurcula MT767744
Decma fissa NC 033981
Pseudocosmetura anjiensis NC 033853
Acosmetura nigrogeniculata NC 045212
Xizicus howardi KY458226
Alloxiphidiopsis emarginata NC 065298
Grigoriora cheni OM892710
Grigoriora cheni NC 068776
Microconema sp 1 LLL 2022a OM937110
Microconema clavata MT849272
Xizicus fascipes OM892714
Xizicus fascipes NC 018765
Xizicus maculatus NC 040974
Xiphidiopsis gurneyi NC 039981
Chandozhinskia hastaticercus OM892711
Chandozhinskia hastaticercus NC 068777
Phlugiolopsis punctata OM892713
Phlugiolopsis punctata OM892709
Phlugiolopsis punctata NC 068775
Phlugiolopsis tuberculata OM892715
Phlugiolopsis tuberculata NC 068779
Phlugiolopsis brevis OM913480
Phlugiolopsis brevis NC 068752
Paraphlugiolopsis jiangi OM892712
Paraphlugiolopsis jiangi NC 068778
