# Supplementary material for: Comparative Mitogenomics and Phylogenetic Implications for Nine Species of the Subfamily Meconematinae (Orthoptera: Tettigoniidae)
Source: Insects. 2024 Jun 3;15(6):413. doi: 10.3390/insects15060413 (PMC11204050; doi:10.3390/insects15060413)
Supplement: Supplementary file 1 [file insects-15-00413-s001.zip › Table S1.The nucleotide composition of complete mitochondrial genome of 9 species of the subfamily.docx]

**Table S1.** The nucleotide composition of complete mitochondrial genome of 9 species of the subfamily Meconematinae.

| Species | Region | T% | C% | A% | G% | A+T% | AT-Skew | GC-Skew |
| --- | --- | --- | --- | --- | --- | --- | --- | --- |
| *Phlugiolopsis tribranchis* | Genome | 33.14 | 19.92 | 35.86 | 11.08 | 68.99 | 0.0394 | -0.2852 |
|  | PCGs | 40.47 | 15.62 | 28.89 | 15.01 | 69.37 | -0.1669 | -0.0201 |
|  | tRNAs | 36.35 | 10.84 | 38 | 14.81 | 74.35 | 0.0221 | 0.1551 |
|  | rRNAs | 38.49 | 7.62 | 35.3 | 18.58 | 73.8 | -0.0433 | 0.4182 |
|  | CR | 30.07 | 26.73 | 30.71 | 12.49 | 60.78 | 0.0105 | -0.3633 |
| *Grigoriora cheni* | Genome | 36.37 | 16.53 | 37.24 | 9.86 | 73.61 | 0.0117 | -0.2526 |
|  | PCGs | 42.16 | 13.66 | 30.68 | 13.5 | 72.84 | -0.1575 | -0.0059 |
|  | tRNAs | 37.2 | 10.98 | 37.95 | 13.86 | 75.15 | 0.01 | 0.116 |
|  | rRNAs | 39 | 7.54 | 36.32 | 17.14 | 75.32 | -0.0355 | 0.3888 |
|  | CR | 39.29 | 17.48 | 35.4 | 7.83 | 74.69 | -0.0521 | -0.3811 |
| *Chandozhinskia hastaticerca* | Genome | 33.89 | 18.55 | 37.11 | 10.45 | 71 | 0.0453 | -0.2793 |
|  | PCGs | 41.01 | 14.94 | 29.6 | 14.46 | 70.61 | -0.1616 | -0.0164 |
|  | tRNAs | 36.44 | 10.64 | 38.23 | 14.69 | 74.67 | 0.0239 | 0.1599 |
|  | rRNAs | 38.88 | 7.91 | 35.92 | 17.29 | 74.8 | -0.0395 | 0.3724 |
|  | CR | 27.28 | 30.72 | 33.21 | 8.78 | 60.5 | 0.098 | -0.5556 |
| *Microconema* sp. | Genome | 33.41 | 19.21 | 37.12 | 10.26 | 70.53 | 0.0527 | -0.3035 |
|  | PCGs | 39.65 | 15.79 | 29.41 | 15.15 | 69.06 | -0.1484 | -0.0208 |
|  | tRNAs | 36.15 | 10.56 | 38 | 15.29 | 74.14 | 0.025 | 0.183 |
|  | rRNAs | 38.54 | 7.78 | 36.02 | 17.66 | 74.56 | -0.0337 | 0.3881 |
|  | CR | 34.31 | 22.09 | 37.23 | 6.37 | 71.54 | 0.0409 | -0.5524 |
| *Paraphlugiolopsis jiangi* | Genome | 34.45 | 18.39 | 36.6 | 10.56 | 71.05 | 0.0302 | -0.2706 |
|  | PCGs | 41.06 | 14.94 | 29.82 | 14.19 | 70.87 | -0.1586 | -0.0257 |
|  | tRNAs | 36.38 | 10.91 | 38.01 | 14.7 | 74.39 | 0.0219 | 0.1481 |
|  | rRNAs | 38.41 | 7.68 | 35.59 | 18.32 | 74 | -0.038 | 0.4092 |
|  | CR | 32.82 | 24.3 | 31.35 | 11.53 | 64.16 | -0.0229 | -0.3564 |
| *Phlugiolopsis punctata* | Genome | 33.97 | 19.03 | 36.01 | 11 | 69.98 | 0.0293 | -0.2674 |
|  | PCGs | 40.08 | 16.08 | 29.41 | 14.42 | 69.5 | -0.1535 | -0.0544 |
|  | tRNAs | 36.36 | 10.86 | 37.87 | 14.91 | 74.23 | 0.0204 | 0.1573 |
|  | rRNAs | 38.77 | 7.75 | 35.54 | 17.94 | 74.31 | -0.0435 | 0.3963 |
|  | CR | 34.16 | 22.09 | 31.29 | 12.46 | 65.45 | -0.0439 | -0.2787 |
| *Phlugiolopsis brevis* | Genome | 34.4 | 18.46 | 36.93 | 10.21 | 71.33 | 0.0354 | -0.2877 |
|  | PCGs | 41.02 | 15.17 | 29.72 | 14.09 | 70.74 | -0.1598 | -0.0369 |
|  | tRNAs | 36.42 | 10.97 | 37.79 | 14.81 | 74.21 | 0.0185 | 0.1489 |
|  | rRNAs | 38.55 | 7.63 | 35.78 | 18.03 | 74.33 | -0.0372 | 0.4052 |
|  | CR | 34.13 | 21.52 | 34.98 | 9.37 | 69.11 | 0.0123 | -0.3934 |
| *Xizicus fascipes* | Genome | 33.29 | 19.07 | 36.69 | 10.95 | 69.98 | 0.0487 | -0.2704 |
|  | PCGs | 40.19 | 15.36 | 29.36 | 15.08 | 69.56 | -0.1557 | -0.0094 |
|  | tRNAs | 36.79 | 10.91 | 37.54 | 14.76 | 74.33 | 0.0102 | 0.1497 |
|  | rRNAs | 39.33 | 7.62 | 34.95 | 18.1 | 74.29 | -0.059 | 0.4074 |
|  | CR | 33.08 | 22.93 | 31.42 | 12.57 | 64.5 | -0.0258 | -0.292 |
| *Phlugiolopsis tuberculata* | Genome | 32.99 | 19.65 | 36.76 | 10.6 | 69.75 | 0.054 | -0.2991 |
|  | PCGs | 40.06 | 16.02 | 29.21 | 14.72 | 69.27 | -0.1566 | -0.0423 |
|  | tRNAs | 36.13 | 11.01 | 37.99 | 14.87 | 74.12 | 0.0251 | 0.1489 |
|  | rRNAs | 38.92 | 7.48 | 34.59 | 19.01 | 73.51 | -0.059 | 0.4353 |
|  | CR | 28.77 | 26.13 | 35.93 | 9.17 | 64.7 | 0.1107 | -0.4805 |
