# Supplementary material for: Comparative Mitogenomics and Phylogenetic Implications for Nine Species of the Subfamily Meconematinae (Orthoptera: Tettigoniidae)
Source: Insects. 2024 Jun 3;15(6):413. doi: 10.3390/insects15060413 (PMC11204050; doi:10.3390/insects15060413)
Supplement: Supplementary file 1 [file insects-15-00413-s001.zip › Table S2. 20 sequences were used for KaíóKs value comparison of the subfamily Meconematinae.docx]

**Table S2.** 20 sequences were used for Ka/Ks value comparison of the subfamily Meconematinae, of which 8 were sequenced with the latest sequencing and 12 were downloaded from the GenBank database.

| **Subfamily** | **Wing type** | **Species** | **Accession number** |
| --- | --- | --- | --- |
| Meconematinae | Macropterous | *Xizicus maculatus* | MG779499 |
|  |  | *Decma fissa* | KX057710 |
|  |  | *Microconema clavata* | MT849272 |
|  |  | *Nipponomeconema sinica* | MK903580 |
|  |  | *Xizicus howardi** | KY458226 |
|  |  | *Pseudokuzicus pieli* | KX057712 |
|  |  | *Teratura megafurcula* | MT767744 |
|  |  | *Xiphidiopsis gurneyi** | NC 039981 |
|  |  | *Shoveliteratura triangula* | NC 048466 |
|  |  | *Grigoriora cheni** | OM892710 (This study) |
|  |  | *Microconema* sp. | OM937110 (This study) |
|  |  | *Xizicus fascipes* | OM892714 (This study) |
|  | Brachypterous | *Chandozhinskia hastaticercus* | OM892711 (This study) |
|  |  | *Phlugiolopsis tribranchis* | OM892709 (This study) |
|  |  | *Paraphlugiolopsis jiangi* | OM892712 (This study) |
|  |  | *Phlugiolopsis punctata* | OM892713 (This study) |
|  |  | *Phlugiolopsis brevis* | OM913480 (This study) |
|  |  | *Phlugiolopsis tuberculata* | OM892715 (This study) |
|  |  | *Acosmetura nigrogeniculata* | MK801775 |
|  |  | *Pseudocosmetura anjiensis* | KX057711 |
