# Supplementary material for: Comparative Mitogenomics and Phylogenetic Implications for Nine Species of the Subfamily Meconematinae (Orthoptera: Tettigoniidae)
Source: Insects. 2024 Jun 3;15(6):413. doi: 10.3390/insects15060413 (PMC11204050; doi:10.3390/insects15060413)
Supplement: Supplementary file 1 [file insects-15-00413-s001.zip › Table S3.Substitution saturation tests of different datasets.docx]

**Table S3.** Substitution saturation tests of different datasets.

| **Data partition** | **NumOTU** | **Iss** | **Iss. cSym** | **Psym** | **Iss. cAsym** | **Psym** |
| --- | --- | --- | --- | --- | --- | --- |
| PCG123 | 32 | 0.439 | 0.818 | 0.0000 | 0.572 | 0.0000 |
| PCG123 + rRNAs | 32 | 0.426 | 0.819 | 0.0000 | 0.573 | 0.0000 |
| PCG12 | 32 | 0.436 | 0.819 | 0.0000 | 0.572 | 0.0000 |
| PCG12 + rRNAs | 32 | 0.425 | 0.819 | 0.0000 | 0.573 | 0.0000 |
