# Supplementary figures and images for: Comparative Mitogenomics and Phylogenetic Implications for Nine Species of the Subfamily Meconematinae (Orthoptera: Tettigoniidae)
Source: Insects. 2024 Jun 3;15(6):413. doi: 10.3390/insects15060413 (PMC11204050; doi:10.3390/insects15060413)

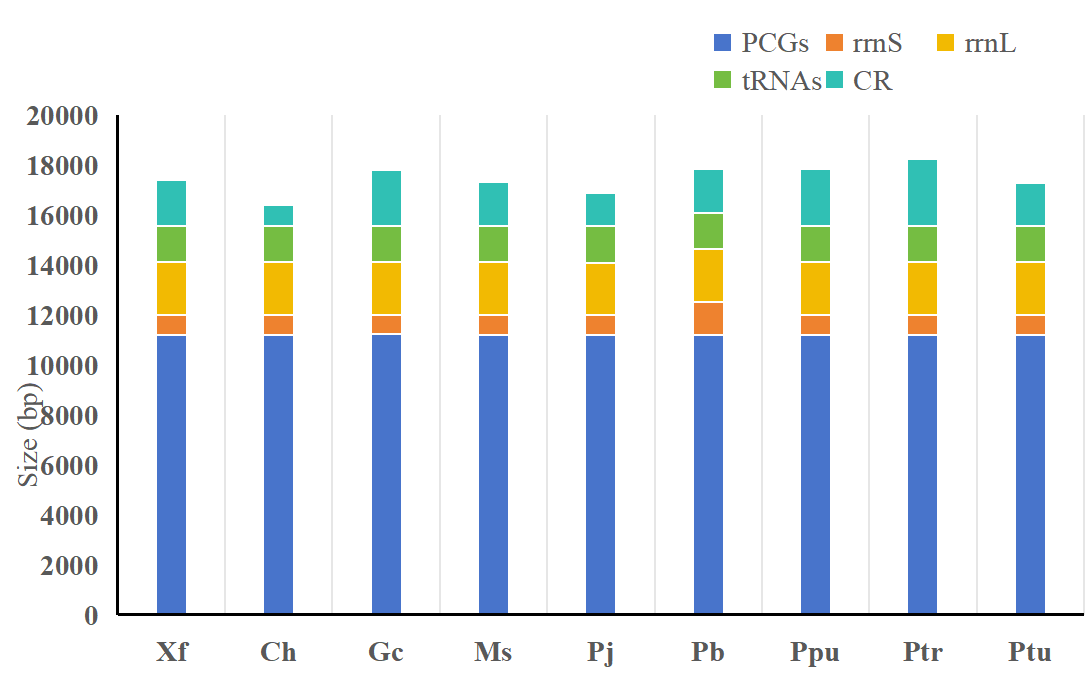

Supplement: Supplementary file 1 [file insects-15-00413-s001.zip › Image files/Figure 1.Size comparison of protein-coding genes (PCGs), transfer RNA genes (tRNAs), rrnL,.png]

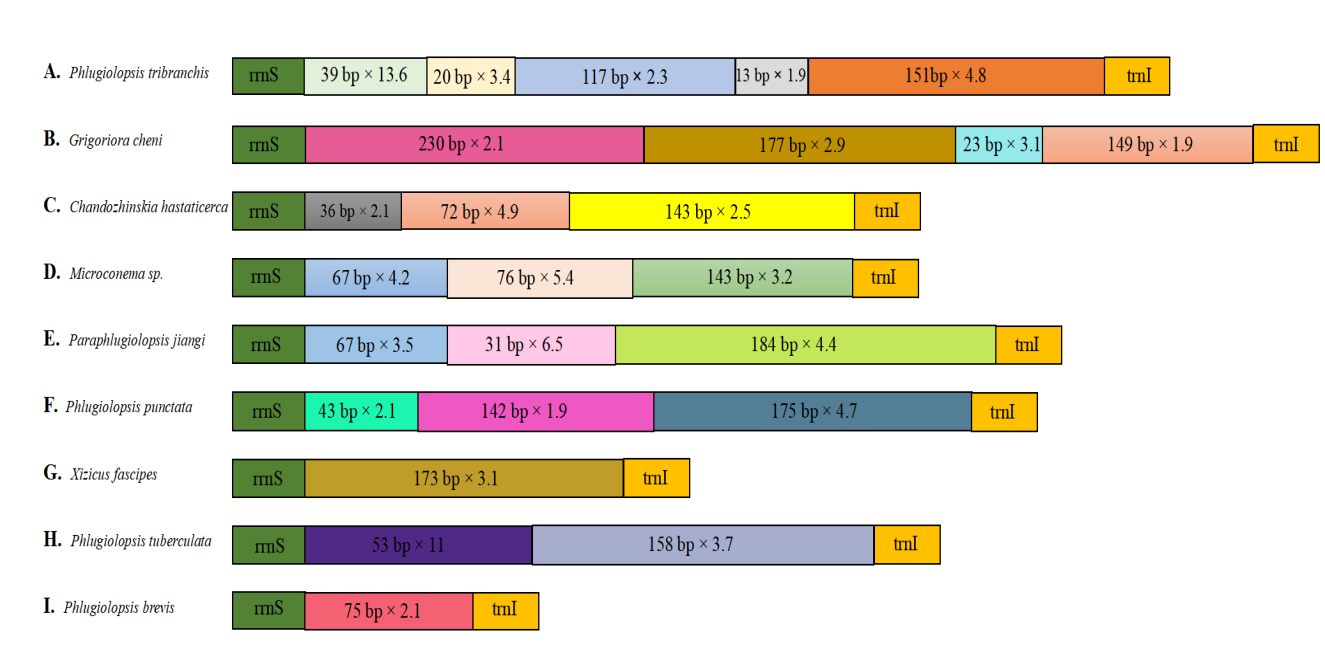

Supplement: Supplementary file 1 [file insects-15-00413-s001.zip › Image files/Figure 2.Schematic diagram of tandem repeat arrangements in the CR.png]

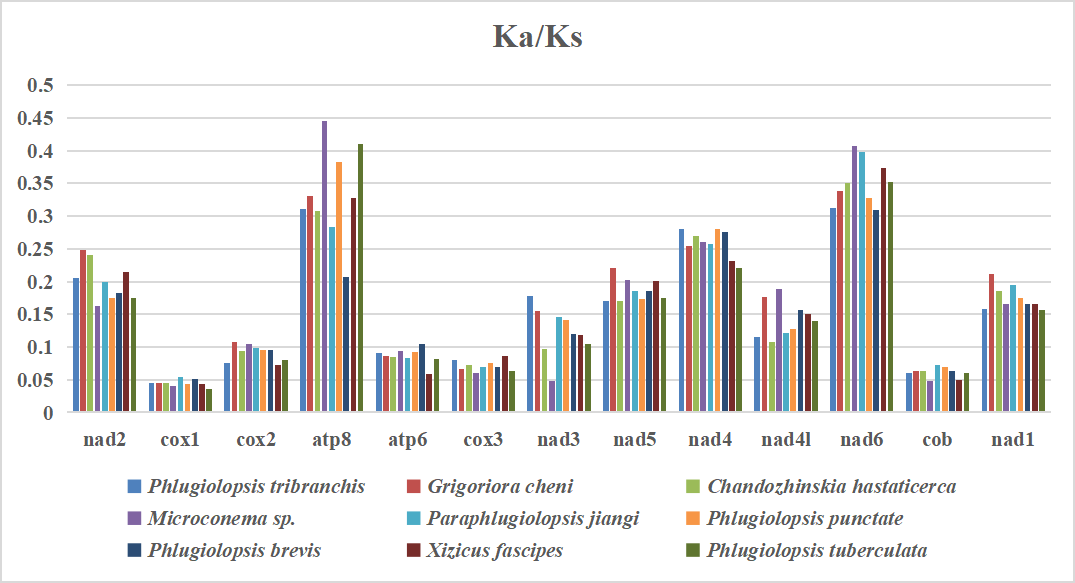

Supplement: Supplementary file 1 [file insects-15-00413-s001.zip › Image files/Figure 3. Ka Ks values of 13 PCGs in nine species of Meconematinae.png]

Tree scale: 0.1

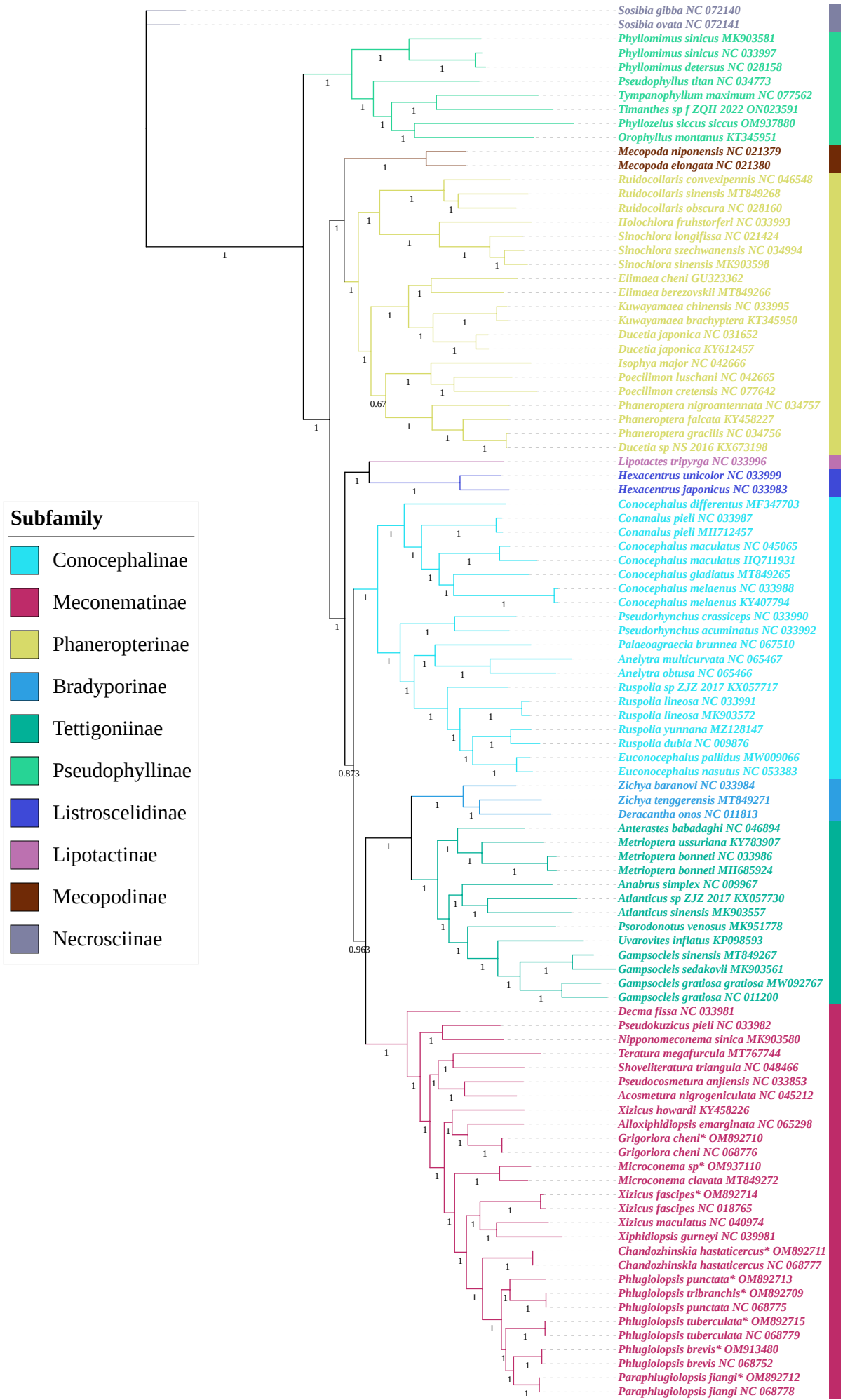

Supplement: Supplementary file 1 [file insects-15-00413-s001.zip › Image files/Figure 4.Phylogenetic tree obtained from BI analysis based on 13 PCGs.pdf]

Tree scale: 1

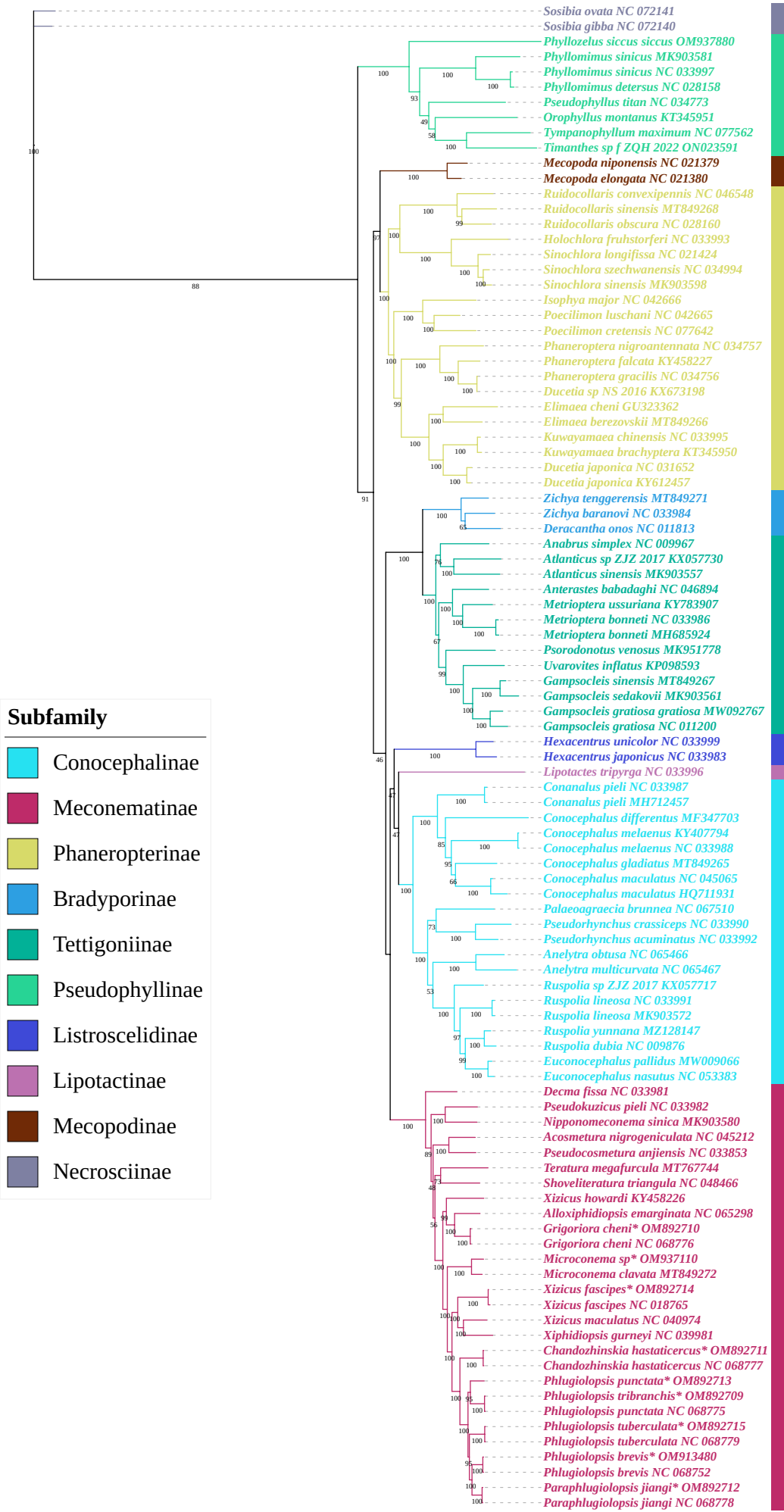

Supplement: Supplementary file 1 [file insects-15-00413-s001.zip › Image files/Figure 5. Phylogenetic tree obtained from ML analysis based on 13 PCGs.pdf]

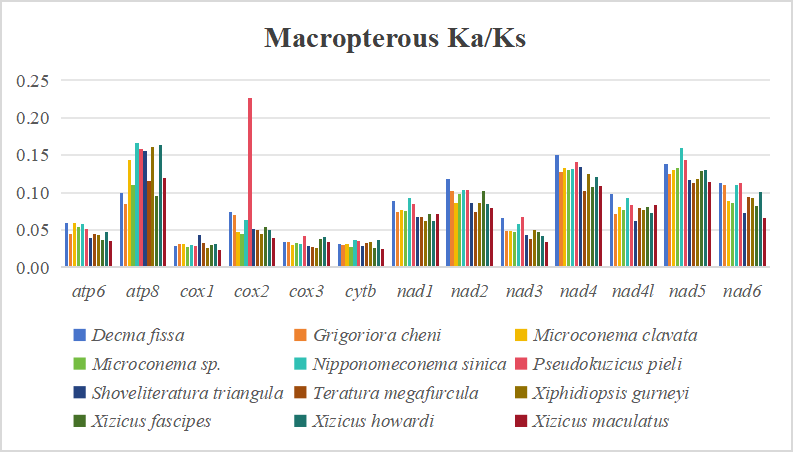

Supplement: Supplementary file 1 [file insects-15-00413-s001.zip › Schedule/Figure S3. KaKs values of 13 PCGs in the macropterous of Meconematinae .png]

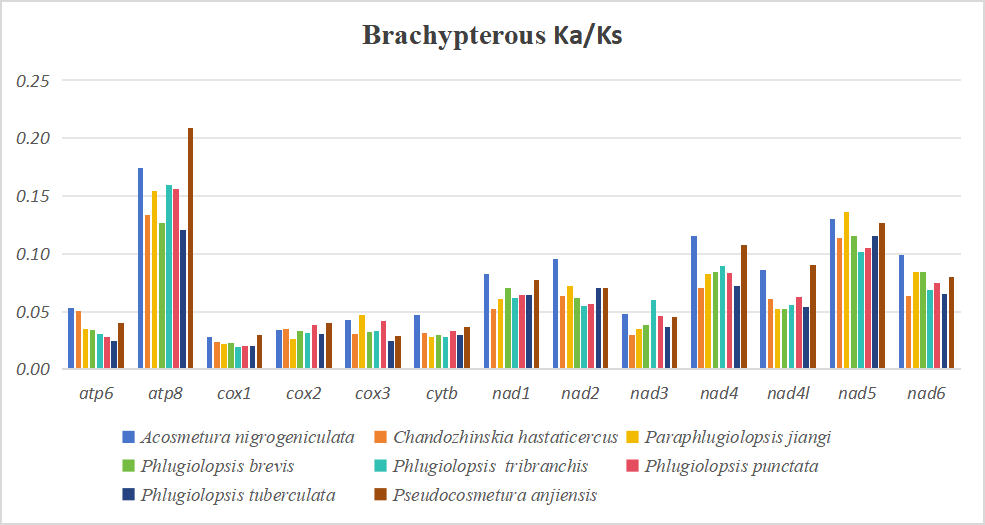

Supplement: Supplementary file 1 [file insects-15-00413-s001.zip › Schedule/Figure S4. KaKs values of 13 PCGs in the brachypterous of Meconematinae.png]

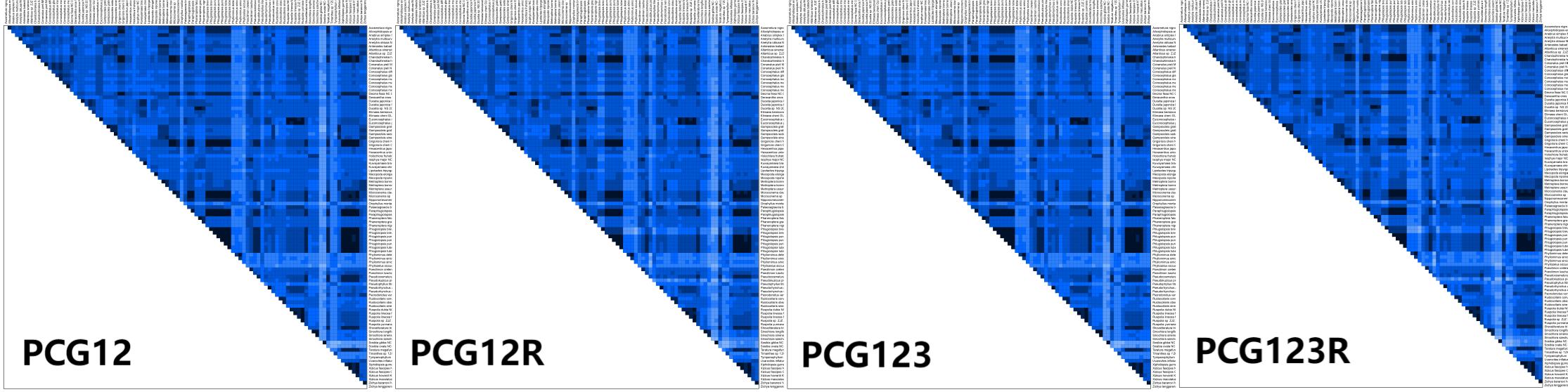

Supplement: Supplementary file 1 [file insects-15-00413-s001.zip › Schedule/Figure S5. Heterogeneity of the sequence composition of the mitochondrial genomes in differen.jpg]

Tree scale: 1

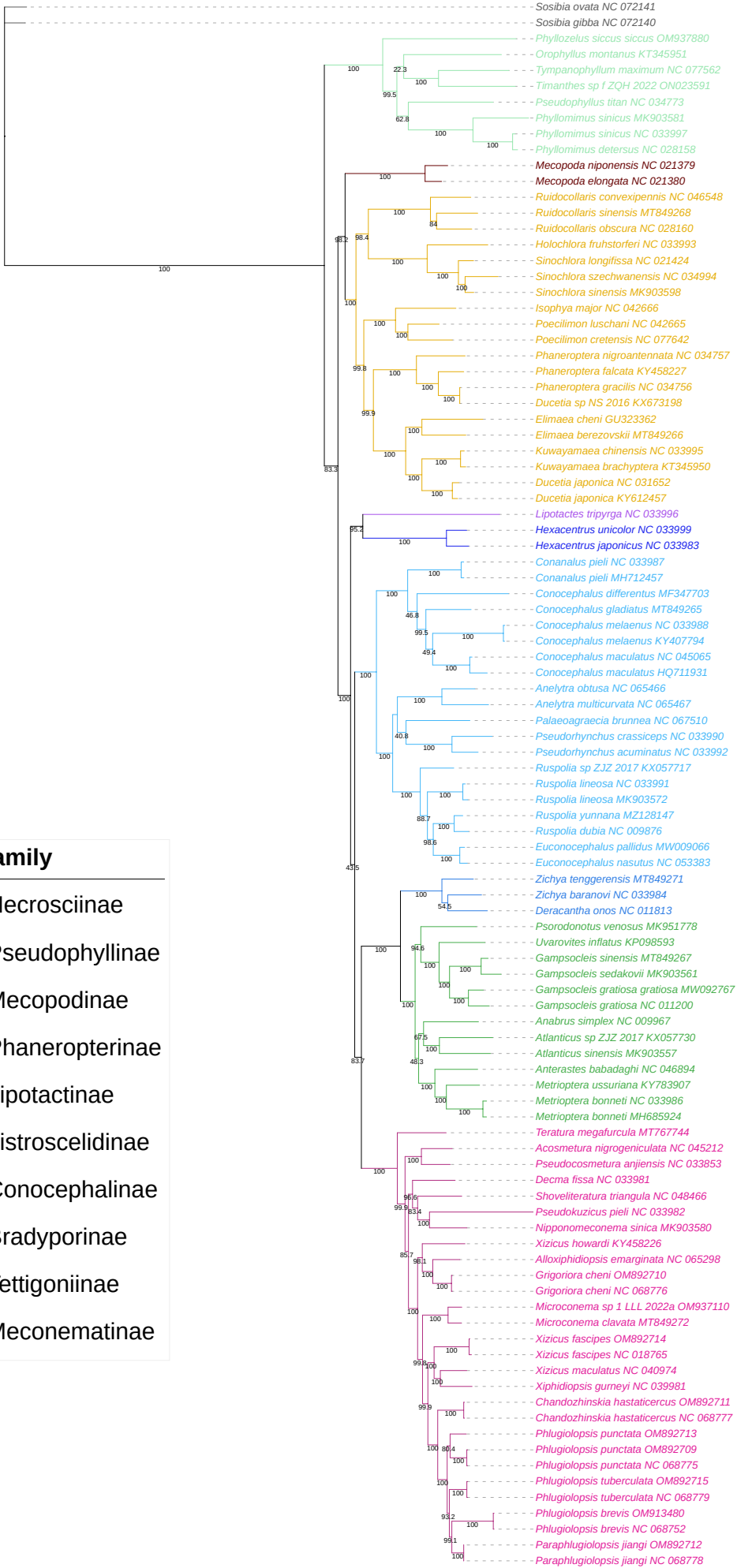

Supplement: Supplementary file 1 [file insects-15-00413-s001.zip › Schedule/Figure S7. Phylogenetic tree obtained from ML analysis based on 13 PCGs + 2rRNA.pdf]
